# Standardized examinees: development of a new tool to evaluate factors influencing OSCE scores and to train examiners

**DOI:** 10.3205/zma001333

**Published:** 2020-06-15

**Authors:** Petra Zimmermann, Martina Kadmon

**Affiliations:** 1 Ludwig-Maximilians-Universität München, Klinikum der Universität, Klinik für Allgemein-, Viszeral- und Transplantationschirurgie, München, Germany; 2 Universität Augsburg, Medizinische Fakultät, Gründungsdekanat, Augsburg, Germany

**Keywords:** OSCE, OSPE, examiner training, quality assurance, standardized examinees

## Abstract

**Introduction: **The Objective Structured Clinical Examination (OSCE) is an established format for practical clinical assessments at most medical schools and discussion is underway in Germany to make it part of future state medical exams. Examiner behavior that influences assessment results is described. Erroneous assessments of student performance can result, for instance, from systematic leniency, inconsistent grading, halo effects, and even a lack of differentiation between the tasks to be performed over the entire grading scale. The aim of this study was to develop a quality assurance tool that can monitor factors influencing grading in a real OSCE and enable targeted training of examiners.

**Material, Methods and Students:** Twelve students at the Medical Faculty of the University of Heidelberg were each trained to perform a defined task for a particular surgical OSCE station. Definitions were set and operationalized for an excellent and a borderline performance. In a simulated OSCE during the first part of the study, the standardized student performances were assessed and graded by different examiners three times in succession; video recordings were made. Quantitative and qualitative analysis of the videos was also undertaken by the study coordinator.

In the second part of the study, the videos were used to investigate the examiners’ acceptance of standardized examinees and to analyze potential influences on scoring that stemmed from the examiners’ experience.

**Results:** In the first part of the study, the OSCE scores and subsequent video analysis showed that standardization for defined performance levels at different OSCE stations is generally possible. Individual deviations from the prescribed examinee responses were observed and occurred primarily with increased complexity of OSCE station content.

In the second part of the study, inexperienced examiners assessed a borderline performance significantly lower than their experienced colleagues (13.50 vs. 15.15, p=0.035). No difference was seen in the evaluation of the excellent examinees. Both groups of examiners graded the item “ocial competence” – despite identical standardization – significantly lower for examinees with borderline performances than for excellent examinees (4.13 vs. 4.80, p<0.001).

**Conclusion:** Standardization of examinees for previously defined performance levels is possible, making a new tool available in future not only for OSCE quality assurance, but also for training examiners. Detailed preparation of the OSCE checklists and intensive training of the examinees are essential.

This new tool takes on a special importance if standardized OSCEs are integrated into state medical exams and, as such, become high-stakes assessments.

## Zusammenfassung

**Einleitung: **Objective Structured Clinical Examinations (OSCE) sind als Format für klinisch-praktische Prüfungen an den meisten medizinischen Fakultäten etabliert und sollen in Zukunft auch in die humanmedizinischen Staatsprüfungen integriert werden. Einflüsse auf die Prüfungsergebnisse durch Prüferverhalten sind beschrieben. Fehlbeurteilungen der studentischen Leistungen resultieren beispielsweise durch systematische Nachsicht, durch Inkonsistenz in der Beurteilung, durch Halo-Effekte oder auch durch fehlende Differenzierung von Leistungen über die gesamte Bewertungsskala. Ziel der vorliegenden Arbeit war es ein Qualitätssicherungsinstrument zu entwickeln, das zukünftig die Überprüfung von Einflussfaktoren auf Bewertungen in einem realen OSCE ebenso wie eine gezielte Prüfer-Schulung ermöglicht.

**Material, Methoden und Studierende: **Zwölf Studierende der Medizinischen Fakultät Heidelberg wurden trainiert, eine definierte Leistung für jeweils eine chirurgische OSCE-Station zu erbringen. Es wurde ein Niveau für eine exzellente und eine Borderline-Leistung festgelegt und operationalisiert. Im ersten Teil der Studie wurde in einem Überprüfungs-OSCE die standardisierte Leistung dreimal hintereinander mit unterschiedlichen Prüfern/innen überprüft, bewertet und auf Video aufgenommen. Eine zusätzliche quantitative und qualitative Bewertung erfolgte durch die Studienleiterin anhand der Videoanalyse.

Im zweiten Teil der Studie wurden die Videoaufnahmen genutzt um die Akzeptanz für Standardisierte Prüflinge bei Prüfern/innen zu erheben und potentielle Einflüsse auf die Leistungsbewertung durch die Prüfererfahrung zu analysieren.

**Ergebnisse: **Im ersten Teil der Studie zeigten die Bewertungen im OSCE und die nachfolgende Videoanalyse, dass eine Standardisierung für definierte Leistungsniveaus an verschiedenen OSCE-Stationen grundsätzlich möglich ist. Einzelne Abweichungen von den erwarteten Antworten wurden beobachtet und traten vor allem mit zunehmender inhaltlicher Komplexität der OSCE-Station auf.

Im zweiten Studienteil bewerteten unerfahrene Prüfer/innen eine Borderline-Leistung signifikant schlechter als ihre erfahrenen Kolleg/innen (13,50 vs. 15,15, p=0,035). In der Bewertung der „Exzellenten Prüflinge“ zeigte sich kein Unterschied. Beide Prüfergruppen bewerteten das Item „Soziale Kompetenz“ – trotz identischer Standardisierung - bei Prüflingen mit einer Borderline-Leistung signifikant schlechter im Vergleich zu den „Exzellenten Prüflingen“ (4,13 vs. 4,80, p<0,001)

**Schlussfolgerung:** Die Standardisierung von Prüflingen für zuvor definierte Leistungsniveaus ist möglich, wodurch zukünftig ein neues Instrument sowohl zur Qualitätssicherung in OSCE-Prüfungen als auch zur Prüferschulung zur Verfügung steht. Eine detaillierte Vorbereitung der OSCE-Checklisten ebenso wie ein intensives Training mit den Prüflingen sind dabei unerlässlich.

Dieses neue Instrument gewinnt besondere Bedeutung, wenn standardisierte OSCE-Prüfungen in die medizinischen Staatsexamina integriert und somit als high-stakes Examen eingesetzt werden.

## Introduction

The Objective Structured Clinical Examination (OSCE) is an established assessment format at most medical schools and is especially suited for evaluating practical clinical skills [1], [2], [3], [4], [5], [6], [7], [8], [9], [10], [11], [12], [13], [14], [15]. An AMEE guideline defines binding standards and metrics for ensuring the quality of OSCEs [9]. The creation of blueprints for both the exam content and the exam format is recommended for all required assessments. A blueprint mapping out exam content and the corresponding stations for the respective subject areas should also form the basis of an OSCE. Based on the blueprint, checklists are created and critically reviewed, and standards are set for performance expectations. A good reliability and inter-rater reliability can be achieved through a sufficient number of OSCE stations, regular standard setting, adaption of the checklists, and regular examiner training. Test statistical analysis of the results should be used to detect problems with the checklists or examiners and to minimize problems by regularly repeating the process described above [9], [15], [16], [17], [18].

Many studies analyze potential factors that influence OSCE scores. These factors take on particular importance when the assessment format is used for a high-stakes exam, as is currently being discussed in Germany in regard to the state medical examinations [19]. Harasym et al. were able to show that stringency or leniency on the part of the examiners can lead to scores that are systematically too high or too low [13]. The student’s performance level also appears to influence the reliability of the scores given by examiners. Byrne et al. describe that a good student performance was evaluated with more precision than a borderline performance [4]. Yeates et al. determined in several studies that a good performance was graded higher if the performance immediately prior to it was a poor one [7], [20]. At the same time, a borderline performance was assessed lower if the examiner had observed a good performance immediately before. In addition, the effects on grading as a result of halo effects and a lack of differentiation on the entire grading scale have also been described [21]. Schleicher et al. were able to show in a study encompassing multiple medical schools that student performances were assessed differently by local and central examiners. Simultaneously, a trend was seen toward different grading behavior depending on the genders of the examiners and examinees [22].

All previous studies on potential influencing factors and on quality assurance of the test format are based on analyses of results from live observations or videos of OSCEs. Although these analyses are based on OSCEs that, in general, were preceded by a standardized briefing of the examiners, they were, however, subject to potential influences stemming from the examinees and were not standardized, so that, ultimately, examiner characteristics could not be fully isolated for analysis.

A suitable tool does not yet exist to simulate potential influences stemming from the examinee for direct analysis of such influences on examiner behavior and exam results. At the same time, no suitable tool has been available to train examiners in a targeted manner regarding the potential limitations concerning the reliability of grading OSCE performance.

Simulated patients are now an integral part of medical education and medical assessments. They offer an opportunity to practice physician-patient interactions in a safe environment and these patients can play an assigned role in a standardized manner. At the same time, it is possible to vary the individual parameters, e.g. the simulated patient’s reaction or the extent of the disease, to simulate different situations for students [23], [24], [25].

Based on the concept of simulated patients, it was our aim to transfer this concept of standardization to student performance on an OSCE. In the first part of this study, we investigated the possibility of training students to reproduce a defined performance on an OSCE. In the second part, we used the video recordings from the first part to analyze the influence of examiner experience on the grades they assigned for the performances and to evaluate the basic acceptance of standardized examinees by examiners.

As a result, there is a new tool for OSCE quality assurance that also enables the identification of individual factors influencing assessment and the targeted training of examiners in the future.

## Material, methods and students

Twelve students were each trained to perform in a standardized manner at three different stations of the OSCE on surgery at the Medical Faculty of the University of Heidelberg. Per station, two students were taught to give a standardized excellent performance and two students to give a standardized borderline performance; there was one female and one male student for each performance level. A student who had been prepared to give an excellent performance at the OSCE abdominal examination station was unable to participate on short notice for health reasons.

The score for an excellent performance was defined as the maximum number of possible points on the checklist, minus no more than two points; a borderline performance was the required minimum number of points to pass, plus or minus one point (minimal competency).

The lowest passing score for the entire OSCE is the sum total of all minimum competencies on Heidelberg’s surgical OSCE.

**Figure 1:**
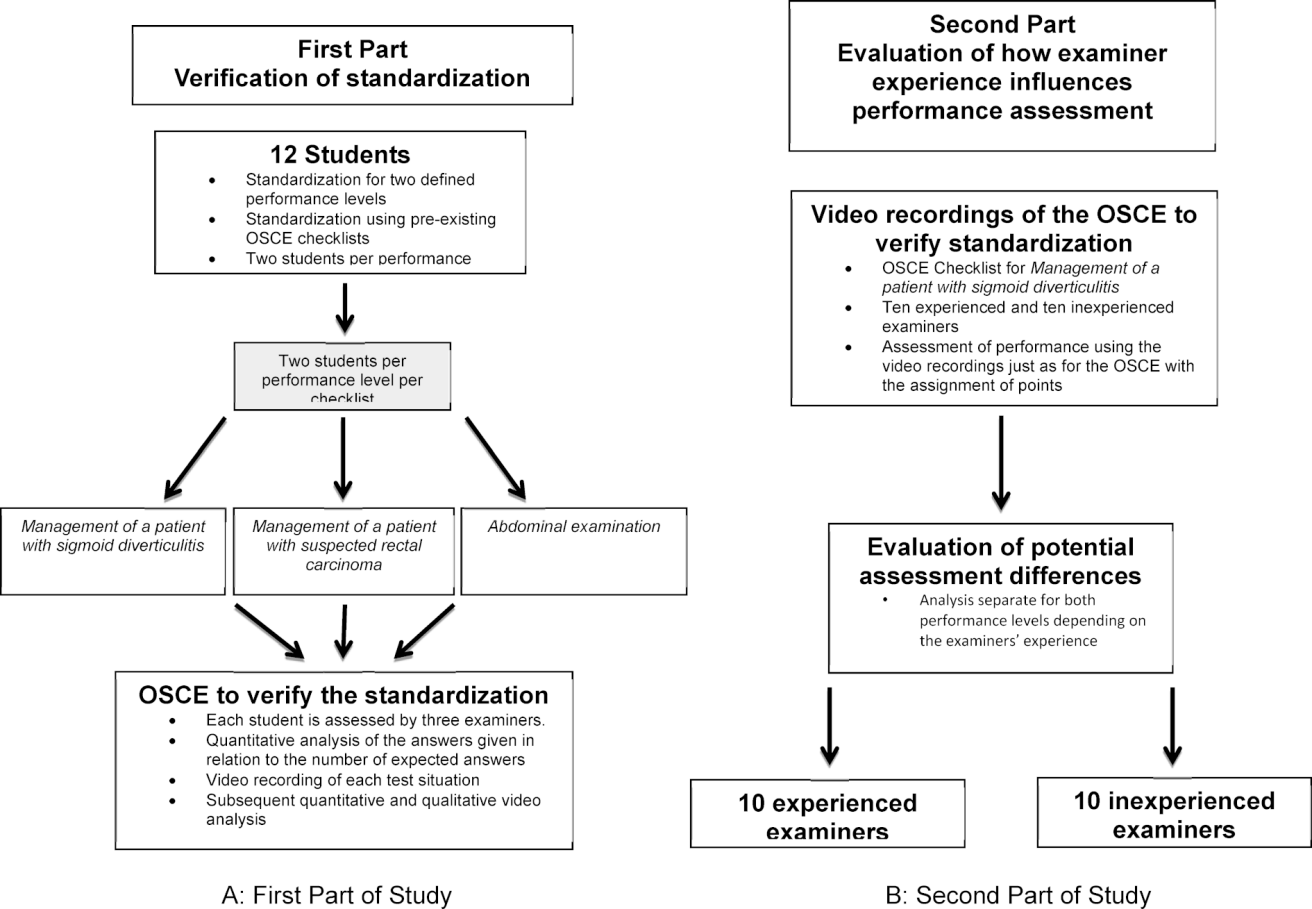
Schematic illustration of the study design with the first and second parts of the study

Figure 1 (Fig. 1) illustrates the study design; figure 1A (Fig. 1) describes the first part of the study and figure 1B (Fig. 1) the second.

### OSCE checklists

Three checklists were selected whose use was already well established in the surgical OSCE and which had undergone repeated internal review. These checklists were for the following OSCE stations:

- Management of a patient with sigmoid diverticulitis;
- Management of a patient with suspected rectal carcinoma;
- Abdominal examination.

All of the checklists had a minimum of 0 and a maximum of 25 points. Each checklist consisted of five items, for which a maximum of five points each could be given. Each item covered a different number of required answers.

Minimal competency was defined as the number of points on a checklist necessary to pass. This also defines the minimum expectancy for each station based on the checklists and is routinely reviewed and defined by way of internal standard setting. The minimal competency for the checklists used was 17 points.

The maximum length of time for the exam was nine minutes per checklist; one minute was given to move between stations. The checklists also listed the grading subcategories (e.g. anamnesis, clinical exam, etc.) and the relevant individual items for assigning points:

- 5 points: all items completed without assistance;
- 3 points: all items completed in full with assistance from the examiner;
- 1 point: items were not fully completed despite assistance from the examiner.

It is clear for each graded category whether points should be given globally for overall impression or on the basis of answers to individual items.

Each checklist contains a brief case vignette, a task for each individual item, and the expected answers. Possible questions asked along the way by the examiner are not predefined.

The station checklists for *sigmoid diverticulitis and rectal carcinoma* cover the taking of the standardized patient’s history (item 1), the determination of differential diagnoses based on the case history details (item 2), the decision which suitable diagnostics should be done in the actual situation (item 3), and for the sigmoid diverticulitis station, the description of a CT image from the patient case. Item 4 on both checklists covers the interaction with the standard patient regarding further diagnostic/therapeutic measures. Item 5 evaluates social competence. This also includes the extent to which the students adequately introduce themselves to the patients, how they behave toward the patients, for instance, if they are able to keep eye contact.

The checklist for the abdominal examination station covers the sequential steps to examine a patient with lower abdominal pain on the right side (item 1), checking for signs of peritonitis (item 2), explaining the performance of a digital rectal exam and the findings (item 3), examining the liver (item 4), and examining the spleen (item 5).

#### Modification of the OSCE checklists

To standardize the performance of the standardized examinees and to verify that this performance can be reproduced repeatedly, two new versions of the existing checklists used for the surgical OSCE were generated.

#### Checklists to standardize the examinees

**Figure 2:**
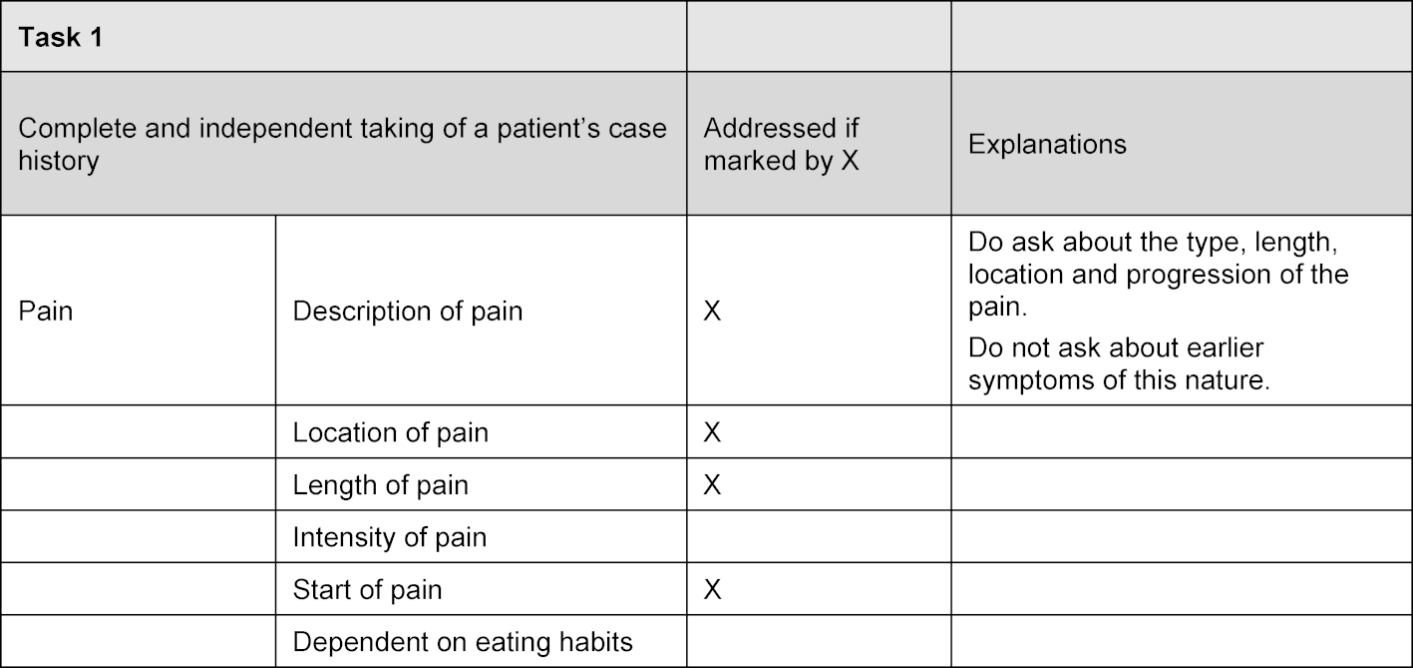
Example of an excerpt from a checklist defining the borderline examinee answers

To standardize the examinees, all of the checklists were operationalized in detail. For the two defined levels of performance, it was determined for each possible answer to a checklist item, whether the examinees should respond with a certain answer or not. In another field it was noted how the examinees should conduct themselves when asked a particular question, e.g. to answer hesitantly or only when prompted (see figure 2 (Fig. 2)).

#### Checklists for evaluating performance

**Figure 3:**
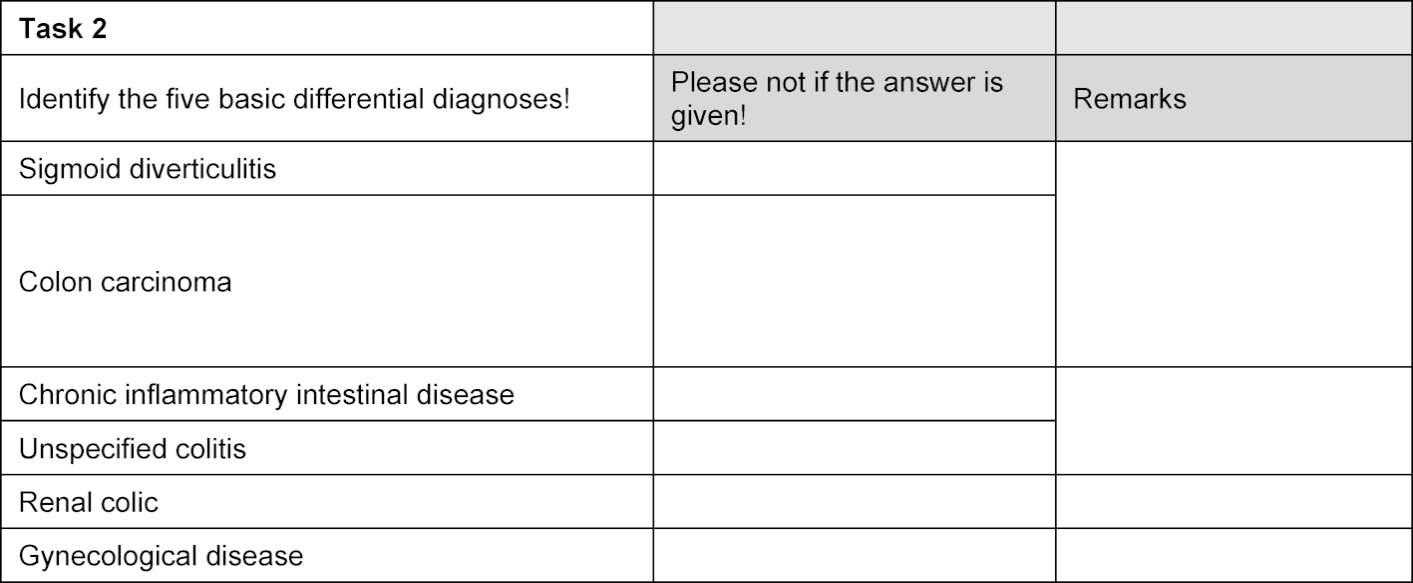
Example of an excerpt from a modified examiner checklist

For the examiner to grade the performance, the evaluation part of the OSCE checklists was modified so that the examiner could note for each possible answer to each task whether or not that answer had been given (see figure 3 (Fig. 3)). To eliminate potential systematic differences in assessment by the examiners, we did not carry out the standardization of the evaluation at the performance level using a global point value for each item, as is done in a real OSCE. A section was added at the end of the checklist in which the examiner was meant to evaluate the performance level using a global grading scale (poor, mediocre, very good) and the authenticity. Concerning the latter, the examiners were asked to evaluate the extent to which they doubted having a real examinee in front of them.

The examiners received the standardized assessment instructions for the surgical OSCE. However, they were instructed not to give any points for the individual items, but rather to tick each possible answer and indicate whether or not it had been given. The examiners were informed only after the OSCE that a standardization of student performance had been undertaken.

#### Standardized students

All 12 students had already completed the Surgery Block and taken the OSCE on surgery. The Surgical Block lasts for one semester and covers the subjects of visceral, vascular, thoracic and heart surgery, urology, orthopedics and trauma surgery, hand and plastic surgery, along with anesthesiology and emergency medicine. Lectures and seminars on pathology and radiology are integrated into the individual subject disciplines.

The students were given the checklists for training. The roles and the expected answers on the modified checklists were discussed in detail with each student. After two weeks to learn the checklists and roles, the test situation was simulated between the students and the study coordinator and corrections were made. As this was done, general challenges were discussed at first and then simulated in real-time as a test situation. Feedback was then given on the necessary changes.

#### First part of the study

##### Process of standardization

In the first part of this study (see figure 1 (Fig. 1), left A), standardization was carried out in a simulated OSCE that was held under real test conditions (time, time to change stations, etc.). The standardized examinees played their roles three times for three different examiners (one male examiner and two female examiners) and were recorded on video. In an additional second step, the videos were analyzed quantitatively and quantitatively by the study coordinator using the modified checklists so that there were six evaluations for each student.

When carrying out the quantitative analysis, the deviations were counted based on the prescribed answers that were supposed to have been given. The instances in which too many or too few answers were given were counted in relation to the correct number of expected answers. The mean percentages of the deviations were calculated for all OSCE run-throughs (3 test situations) and for the quantitative analysis from the subsequent video analysis.

When carrying out the qualitative analysis, the overall impression was evaluated first: The examinee appeared to be authentic (yes/no) and stayed in the standardized role. The following aspects were also evaluated:

- Conduct of the examinee when giving answers (appears confident, unconfident, tends to recite lists);
- Reaction of the examinee to the examiner’s behavior/questions (stays in the role, deviates from the prescribed answers, lets him or herself be forced to give answers);
- Reaction of the examinee to the standard patient’s behavior/questions (stays in the role, deviates from the prescribed answers, lets him or herself be forced to give answers);
- Conduct of the examiners;
- Conduct of the standard patients.

The study coordinator shared responsibility for the organization of the Surgery Block and had acted as an examiner more than 20 times in surgical OSCEs. In addition, she was experienced in the writing of OSCE checklists and exam questions. This study was carried out within the scope of her master’s thesis to attain a Master of Medical Education in Germany (MME-D).

#### Second part of the study

##### Analysis of the influence of examiner experience on performance assessment

In the second part of the study (see figure 1 (Fig. 1), right B), the videos were used to investigate the influence of examiner experience on performance assessment and their acceptance of the standardized examinees. Ten experienced and ten inexperienced examiners watched the video recording of the OSCE station on sigmoid diverticulitis. Experienced examiners had participated at least three times or more in an OSCE and/or had more than five years of clinical experience. Inexperienced examiners were those who had served a maximum of two times as an OSCE examiner and/or had less than five years of clinical experience.

The original checklists from the surgical OSCE administered by the Medical Faculty of Heidelberg University were used to grade performance and required the assignment of one to five points for each item.

A briefing was held to impart general information on administering the test. The following instructions were given:

- The students perform a specific task which must be evaluated. No detailed information was given regarding the performance levels.
- The evaluation must be made based on what is contained in the checklists.
- Five points may only be assigned for a task if all items were accomplished without assistance.
- Three points may only be assigned for a task if all items were fully completed with the assistance of the examiner.
- One point may be given for a task if it was done incompletely despite the assistance of the examiner.
- Stopping and rewinding the video to view it again was not permitted.
- All four test situations must be viewed in sequence and without interruption.

The examiners were only informed after evaluating the videos that the students had been standardized to perform at a defined level.

##### Acceptance of standardized examinees

After evaluating all of the test situations, all of the examiners were surveyed to evaluate the acceptance of standardized examinees and their possible uses. The following was asked directly:

- Assessing the performance was easy for me.
- I would find it easier to assess in a real test situation.
- The assessment of the performance was difficult for me.
- The assessment of performance by good examinees was easy for me.
- The assessment of performance by poor examinees was easy for me.
- I find it makes sense to use standardized examinees to prepare inexperienced examiners.
- Training with video recordings (as opposed to training in a simulated OSCE) is sufficient to prepare examiners.
- Inexperienced examiners should be trained using standardized examinees before conducting real assessments.
- Experienced examiners should simulate test situations using standardized examinees.
- Targeted training of examiners using standardized examinees can make the OSCE objective.
- The performance of the standardized examinees was authentic.

The evaluation was done using a five-point Likert scale with 1=*completely disagree* to 5=*completely agree*.

##### Statistical analysis

Only a purely descriptive and qualitative analysis was carried out for the first part of the study due to the small cohorts and the individual approaches. Further statistical tests were not applied. The OSCE answer sheets were analyzed as to whether too many or too few answers had been given. Later, the study coordinator used the video recordings to analyze which difficulties arose when answering the questions. All of the quantitative analyses based on the OSCE checklists and the secondary video analysis were compiled and the percentages of deviations from the prescribed answers were calculated for all of the evaluations (see table 1 (Tab. 1)).

For the second part of the study, the results of the comparison between experienced and inexperienced examiners are presented as mean values with standard deviation, if not otherwise indicated. The quantitative parameters were analyzed using the two-sided T-test. Categorical variables are given as absolute values. Statistical significance is assumed when the p-value is <0.05. Statistical analysis was carried out using IBM SPSS Statistics 25 software.

## Results

### First part of study: development of the standardized examinees

#### Verification of the standardization – descriptive analysis

An individual evaluation was carried out at the item level for each examinee. The percentage of deviations in the answers given from the expected number of responses was analyzed based on the standardization. In doing this, all of the evaluations, checklists from the OSCE, and the secondary quantitative video analysis by the study coordinator were compiled. The detailed results can be found in table 1 (Tab. 1). Only three examinees were analyzed for the *abdominal examination* checklist since one student was unable to participate in the OSCE for health reasons.

It became clear that especially students with a borderline performance had problems giving the answers correctly. The deviations were more distinct than in the case of the excellent students.

On the checklists covering *sigmoid diverticulitis and rectal carcinoma*, the difficulties were few for the excellent students: They gave a low percentage of too few answers. Larger deviations were seen for the borderline students. The largest deviation occurred for items 3 and 4. These items covered the determination of additional diagnostic and therapeutic measures.

The largest deviation was seen for the station on *abdominal examination* for item 4 by the students giving a borderline performance in the form of a high percentage of missing answers or incorrect performance of medical examination procedures. For this item, the examinees’ examination of the liver was assessed. On this checklist, borderline students showed overall heterogeneous performances with too many and too few answers. Standardized examinees who gave an excellent performance, on the other hand, had a tendency to give too few answers or not to perform individual steps of the medical examination procedure.

##### Assessment of performance by the examiners

All of the examiners, with one exception, had the impression that these were real examinees and indicated they had perceived the standardized examinees as authentic.

The excellent performance was recognized as such in all cases. The borderline performance was assessed as borderline six times; in all other run-throughs, however, it was deemed to be a poor performance.

##### Qualitative analysis via video analysis

The qualitative analysis of the OSCE videos revealed a series of aspects that had a limiting effect on the standardization. The examinees showed a certain tendency to recite the expected answers as if they were memorized lists. This applied more to the excellent examinees than to the borderline ones. Borderline examinees had difficulties staying in their roles particularly for complex items that required drawing on a diagnostic or therapeutic algorithm and not allowing the examiner to push them into giving more than the standardized answers. On the whole, the standardized examinees were able to do this well. At the same time, it was noticed that occasionally the role was over-exaggerated and, for instance, an intentionally hesitant behavior was acted out in a very pronounced manner. As a result, time became tight in individual test situations.

The conduct of the examiners also influenced the students’ acting of their roles and the results of the standardization. As in real assessments, the examiners showed a tendency to repeat questions or give advice on doing individual tasks. Among other things, this increased the difficulty the students faced in consciously not giving answers. Based on the video analysis, it also became clear that one examiner did not award points for answers which were given or examination steps that were performed. In another situation, an examiner evaluated the response of a simulated patient as the answer given by the examinee.

Likewise, it was observed that simulated patients actively influence the assessment by asking their own questions and preventing the students from giving an answer.

#### Second part of the study: influence of examiner experience on performance assessment and acceptance of standardized examinees

##### Influence of examiner experience on performance assessment

Ten experienced and ten inexperienced examiners were included in the study, with one female and nine male examiners forming the experienced group and three female and seven male examiners forming the inexperienced group. All of the examiners assessed all of the standardized examinees in one test situation during the OSCE in the first half of the study. The details regarding examiner experience are presented in table 2 (Tab. 2).

**Figure 4:**
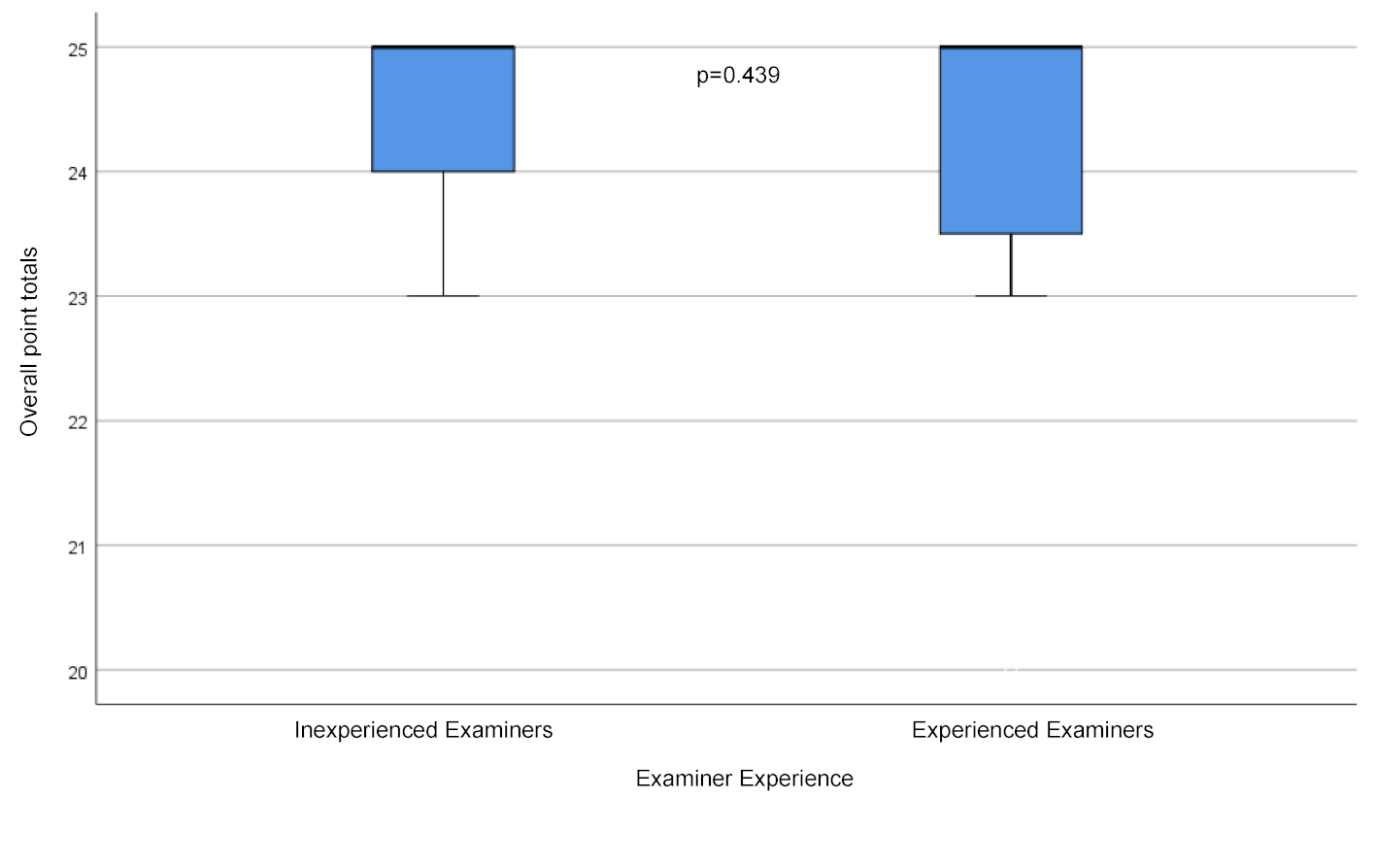
Overall point totals for the students with the excellent performance according the examiners’ experience.

In the assessment of the examinees with excellent performance there was no significant difference between experienced and inexperienced examiners (see table 3 (Tab. 3) and figure 4 (Fig. 4)). In contrast, there was a significant difference between the two groups in their assessments of the borderline examinees. Inexperienced examiners tended to assess the performance lower than their experienced counterparts.

**Figure 5:**
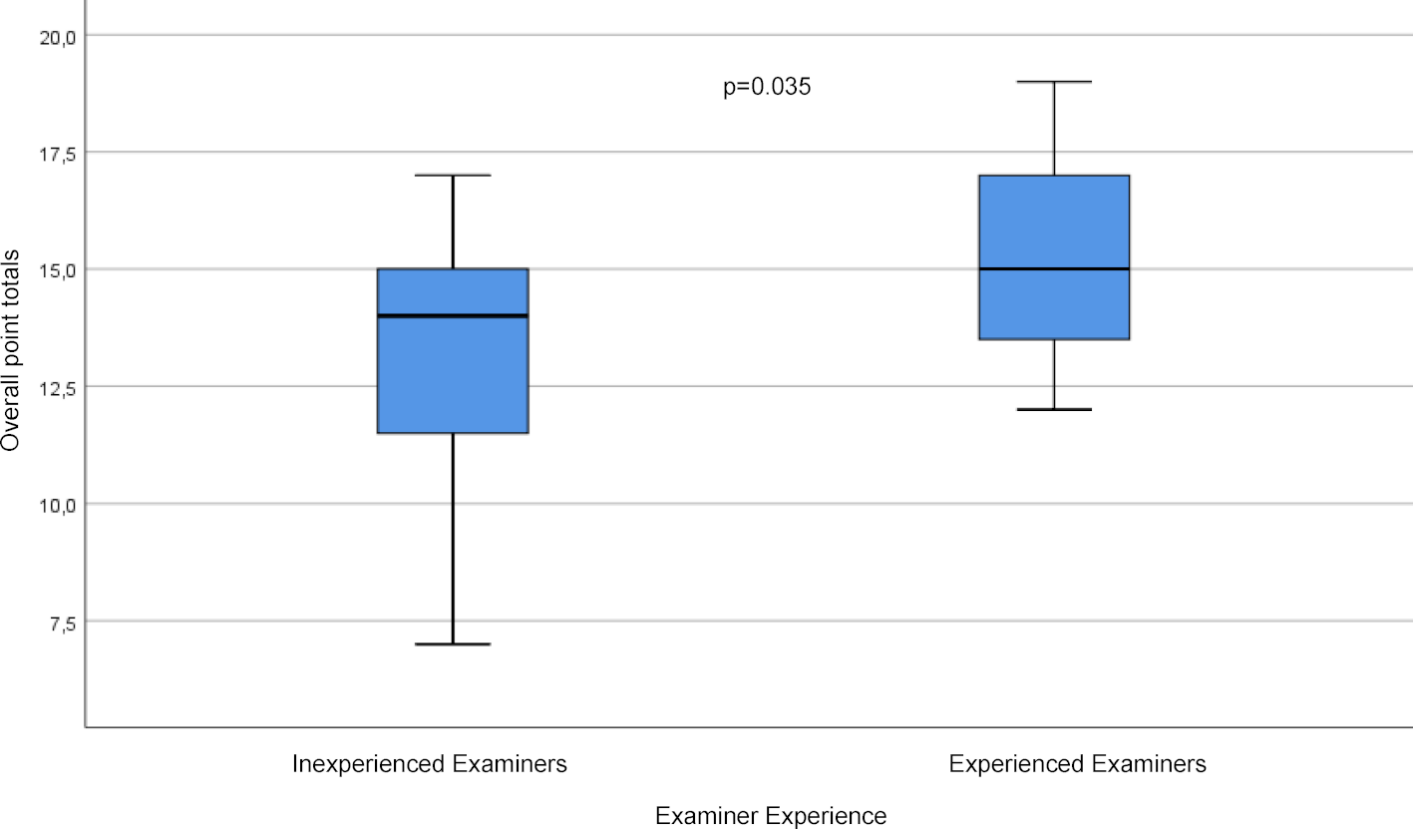
Overall point totals for the students with the borderline performance according to examiners’ experience

Both groups of examiners graded the social competence (item 5), despite identical standardization, lower for the borderline examinees than for the excellent ones (see table 3 (Tab. 3) and figure 5 (Fig. 5)). This difference was statistically significant (4.80 vs. 4.13, p<0.001).

##### Acceptance of standardized examinees

Both groups of examiners perceived the standardized examinees to be authentic and viewed this new tool as an opportunity to make the OSCE even more objective. Both groups found it easier to assess the performance of good students than of borderline students, but still found no difficulties overall in assessing student performance.

**Figure 6:**
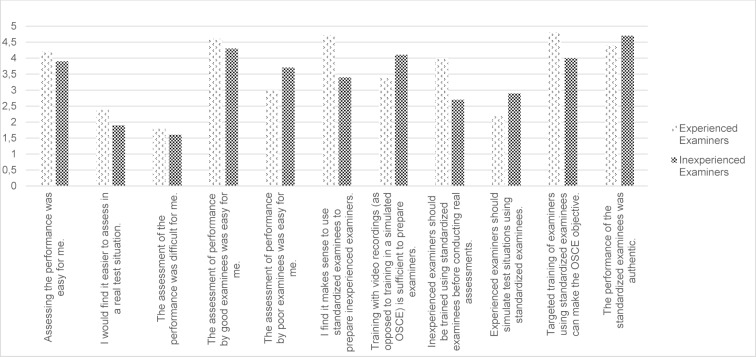
Results of the evaluation questionnaire regarding the standardized students according to experienced and inexperienced OSCE examiners, evaluation using a five-point Likert scale (1=completely disagree, 2=mostly disagree, 3=agree in part, 4=mostly agree, 5=completely agree)

The regular use of standardized examinees to train experienced examiners was favored more by the group of inexperienced examiners than by the experienced group (2.9 vs. 2.0). The detailed results are presented in figure 6 (Fig. 6).

## Discussion

Detailed instructions on how to design, implement and ensure the quality of an OSCE and the resulting good, statistically measured results justify the use of this test format to assess and grade practical clinical skills at medical schools [9], [15], [16], [17], [18]. While OSCEs and OSPEs, to date, have been used primarily as internal university-specific assessments, the current discussion on including them in state medical examinations is making the need for widespread standardization very clear [19]. Despite established quality assurance measures, a variety of studies have been able to show that factors can potentially influence OSCE scores. Such studies often involve extensive staff resources, e.g. independent co-examiners, video analyses, etc. At the same time, it is impossible to eliminate individual influences stemming from examinees and examiners or to standardize these factors satisfactorily. Our aim was to develop a new tool for OSCE quality assurance by applying the concept of standardization to student performance, an approach that enables the identification of individual factors influencing the grading of student performance. Simultaneously, this new tool is also meant to serve as a strategy for training OSCE examiners in the future.

As part of verifying the standardized examinees, it was demonstrated that it is possible to successfully standardize students to meet a previously defined performance level.

The verification of the standardization revealed that deviations occurred in both groups of examinees. Excellent examinees tended more toward giving too few answers and had difficulties not appearing to recite previously memorized lists, while the borderline examinees gave both too few and too many answers. The deviations were overall more distinct for the borderline examinees indicating that the standardization for this performance level is more difficult to achieve.

The answers given by borderline examinees deviated in particular for items in which the description of a diagnostic or therapeutic algorithm was required (see table 1 (Tab. 1)). This suggests that increased complexity of the task makes standardization more difficult. Similar observations were made regarding the complex examination procedures on the abdominal examination checklist where the borderline examinees also deviated from the expected procedural steps (see table 1 (Tab. 1)). In addition to the purely content-based deviations, individual students tended to over-exaggerate their roles.

Both the content-based deviations by the standardized examinees and the different interpretations of the roles they played suggest that the process of standardization itself and specific training for the roles are essential. In the approach followed here, the students were trained using modified checklists on which, depending upon performance level, each possible answer was predefined and rehearsed, whether it was meant to be given or not. From these results it can be understood that the standardization should be trained in even more detail. As is the case when training simulated patients [26], it appears sensible to define a larger role in which the performance level or the characteristic being assessed can be embedded. Since the examiners tended to repeat questions precisely for borderline examinees, the students must be very specially trained for such situations. In particular, attention must be paid to complex tasks and medical examination procedures. Based on the experiences described here, it is wise to let students repeatedly rehearse their roles for verification and to simulate different ways in which examiners intervene in the assessment process to practice conformity with the assigned roles on the part of the standardized examinees. Verifying standardization in a nearly real OSCE is also another option to check if standardization has been satisfactorily achieved. Video recording with subsequent analysis by the trainers and standardized examinees represents an additional training strategy.

An obvious disadvantage of this study is the low case numbers. The study involves one pilot project that is on par with a feasibility study. Future standardization of examinees should take place with more students and in a larger number of test situations than the selected number analyzed here.

In the second part of the study, the video recordings of the OSCE station addressing the *management of a patient* with sigmoid diverticulitis were used for both standardization levels. The extent to which examiner experience affected the evaluation of examinee performance was investigated. This station was used because the standardization for it was the best.

The results of this part of the study show that the two groups of examiners assessed the performance of borderline examinees differently. Inexperienced examiners graded the performance significantly lower and also applied a larger point range to do so. Basically, there are several conceivable explanations for this. Experienced examiners recognize the performance for what it is and correctly classify it as such. On the other hand, this observation could also indicate that experienced examiners do not use the full grading scale for recognizable performance levels and, as described by Iramaneerat, only apply a restricted range of points [19]. At the same time, this result could also be construed as indicating that inexperienced examiners are, under circumstances, less confident in classifying poor performances and thus rate them in a potentially exaggerated manner. A study by Yeates et al. demonstrated that different examiners focus on different aspects when assessing a performance [27]. The results here could therefore be a sign that with increasing clinical or assessment experience, the main focus for assigning points is selected unconsciously. It cannot be fully ruled out that all of the examiners here are not subject to a leniency error that is characterized by a general tendency to rate performances in an extreme manner as poorer or better than they actually are [13]. At the same time, it is possible that the effect described by Yeates et al. is present in that a borderline performance is rated especially poorly if it is observed directly after an excellent performance [7]. In the design selected here, the first and last performances in the video sequence were borderline performances, leaving only one instance where the constellation identified by Yeates et al. could have occurred.

The lower score assigned to social competence for borderline examinees (4.80 vs. 4.13, p<0.001), despite identical standardization and identical performance in the verification of standardization, leaves room to presume a halo effect for both examiner groups. The results of this study suggest that in terms of a halo effect, as described by Iramaneerat et al., the poorer content-based performance leads to a misperception of communication skills [21]. Experienced and inexperienced examiners were affected in equal measure by this, which points out that even having extensive experience as an OSCE assessor cannot negate this effect.

The detected differences in the assessment of borderline examinees depending on the examiner’s experience suggest that this effect could potentially be decisive for passing or failing an OSCE station. The latter makes it clear that targeted examiner preparation is essential, especially if OSCEs are to be used in future state medical exams.

Another question that should be considered and explored in further studies is whether a difference exists in the grading behavior of experienced examiners depending on if they have experience as an OSCE assessor, or only have extensive clinical experience, or both. The experienced examiners in this study all had more than five years of clinical experience, but their experience as OSCE assessor varied between two and more than five times serving as OSCE examiners. This aspect was not pursued further since this study is a pilot project with a low case number.

In this study the use of videos to carry out such an analysis does not, by itself, present a novel approach. It is rather the standardized examinees who offer a possibility in the future to conduct very similar analyses in an OSCE with standardized examinees unconnected to video analyses. It is conceivable that standardized examinees could be included as a “quality standard” in an OSCE. The type of training for standardization must be explored and developed further to minimize deviations. Whether it is possible to standardize a student for several checklists still remains open.

## Conclusions

Standardizing simulated examinees to meet defined performance levels represents a future possibility for directly analyzing influences on the grading behavior of OSCE examiners. Within the scope of high-stakes assessments, especially in regard to the future use of OSCEs in state medical exams, standardized examinees represent, alongside quality assurance, a potential tool to train and prepare OSCE examiners [19].

## Competing interests

The authors declare that they have no competing interests. 

## Figures and Tables

**Table 1:**
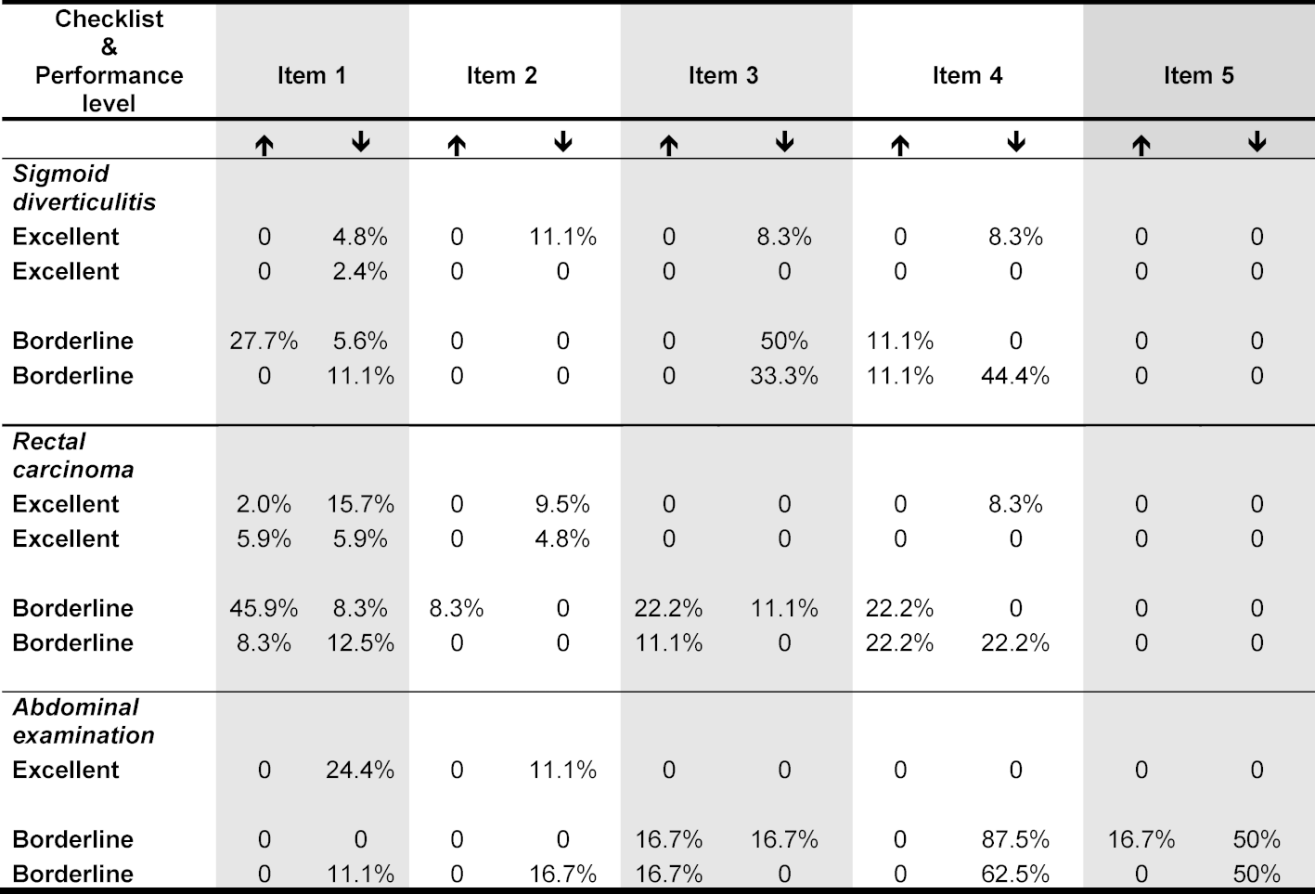
Presented here are the percentages of deviations (too many and too few answers given) from the number of expected answers per item calculated from the quantitative analysis of the OSCE as well as the video analysis differentiated in addition according to standardized performance level (↑ Percent of answers that were given as too many; ↓ Percent of answers that were given as too few). Only three students could be analyzed for the *abdominal examination* checklist because one student was unable to participate in the OSCE for health reasons.

**Table 2:**
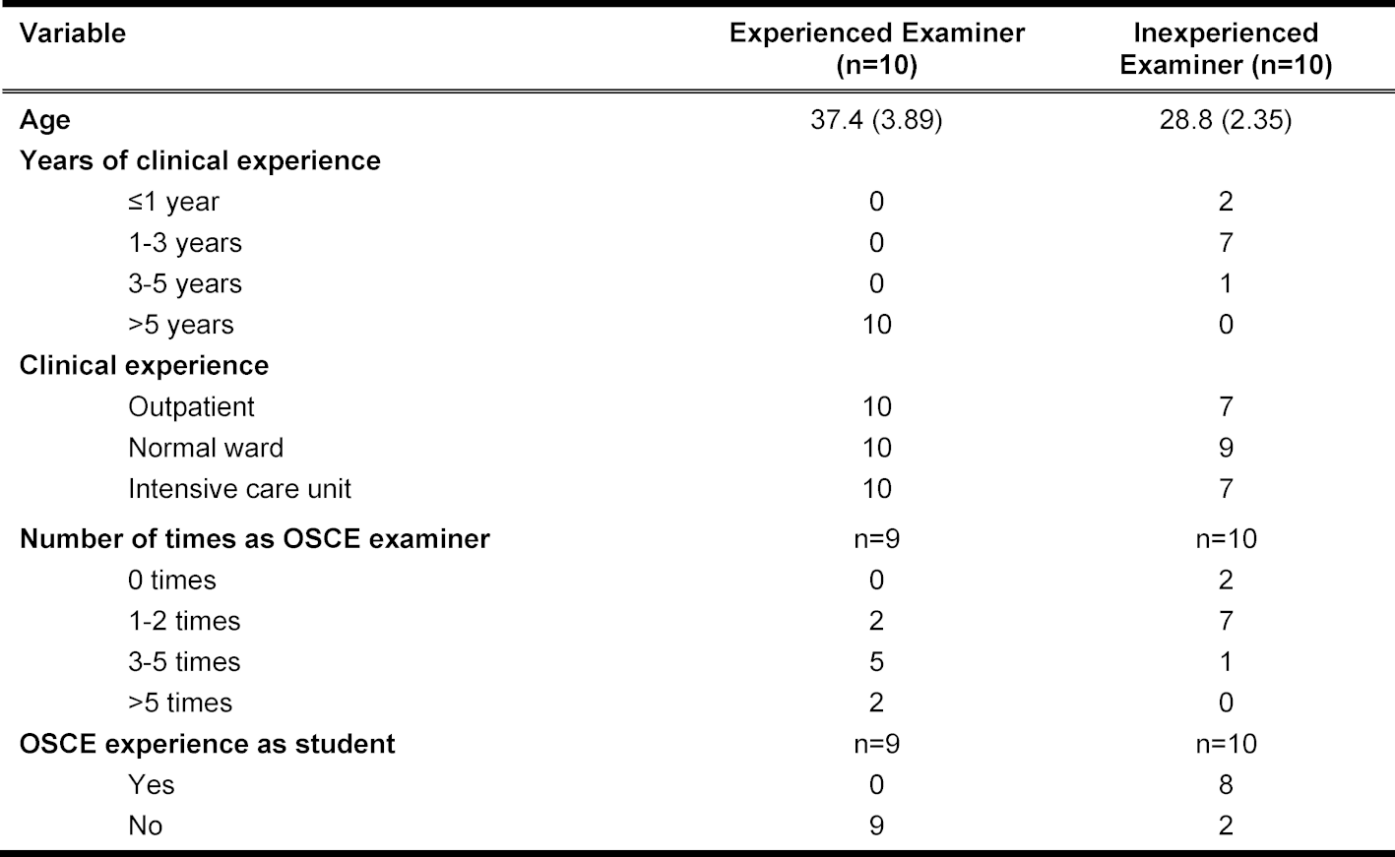
Examiner characteristics, age as mean values with standard deviation; all figures given as absolute values

**Table 3:**
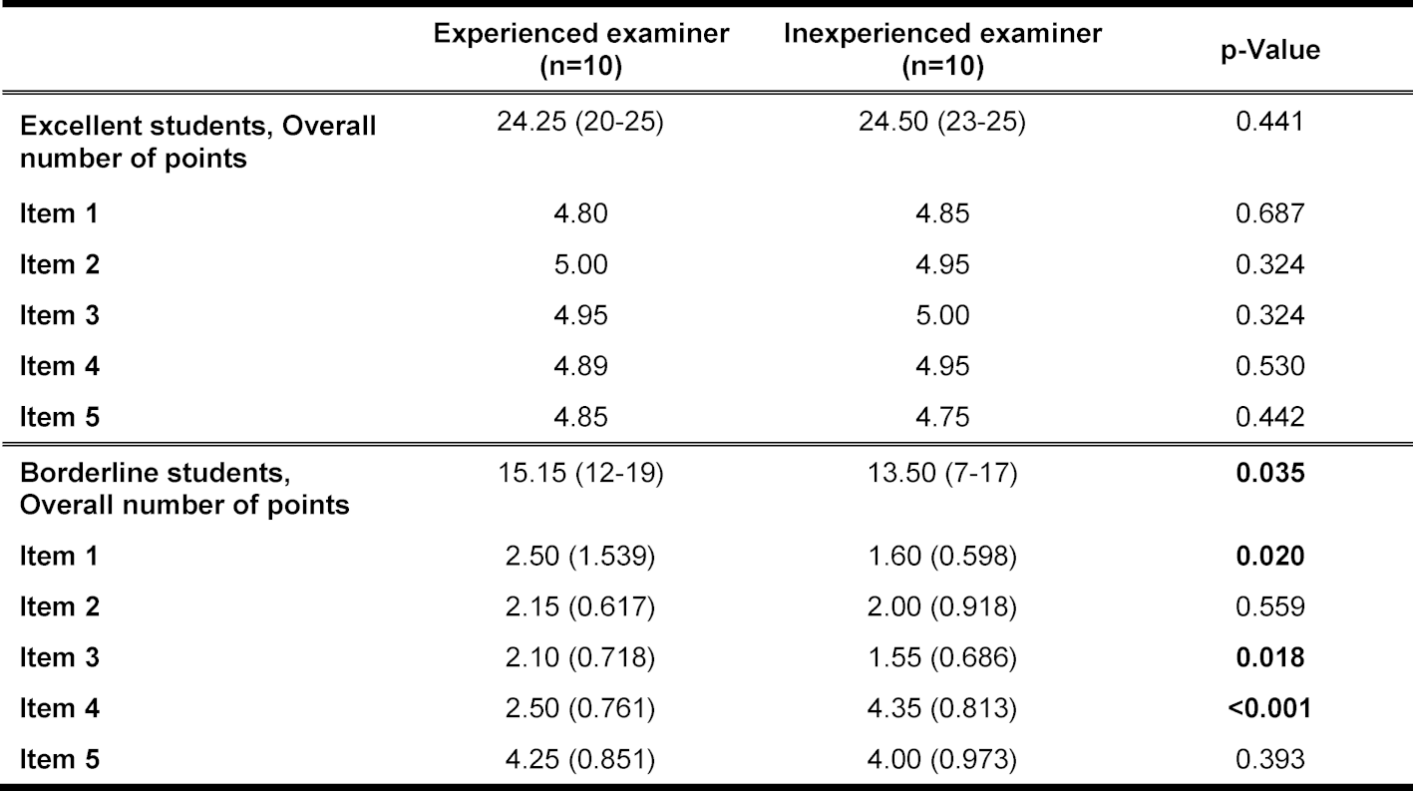
Assessment results for excellent and borderline students according to examiner experience, presented as mean values (min.-max.)

## References

[1] Nikendei C, Kruppa E, Jünger J (2009). Einsatz innovativer Lern- und Prüfungsmethoden an den Medizinische Fakultäten der Bundesrepublik Deutschland- eine aktuelle Bestandsaufnahme. Dtsch Med Wochenschr.

[2] Harden RM, Stevenson M, Downie WW, Wilson GM (1975). Assessment of clinical competence using objective structured examination. Br Med J.

[3] Schleicher I, Leitner K, Jünger J, Möltner A, Rüssler M, Bender B, Sterz J, Stibane T, König S, Frankenhauser S, Kreuder JG (2017). Does quantity ensure quality? Standardized OSCE-stations for outcome-oriented evaluation of practical skills at medical faculties. Ann Anat.

[4] Byrne A, Soskova T, Dawkins J, coombes L (2016). A pilot study of marking accuracy and mental workload as measure of OSCE examiner performance. BMC Med Educ.

[5] Wood TJ, Chan J, Humphrey-Murto S, Pugh D, Touchie C (2017). The influence of first impressions on subsequent ratings within an OSCE station. Adv Health Sci Educ Theory Pract.

[6] Fuller R, Homer M, Pell G, Hallam J (2017). Managing extremes of assessor judgement within the OSCE. Med Teach.

[7] Yeates P, Cardell J, Byrne G, Eva KW (2015). Relatively speaking: contrast effects influence assessors' scores and narrative feedback. Med Educ.

[8] Bartman I, Smee S, Roy M (2013). A method of identifying extreme OSCE examiners. Clin Teach.

[9] Pell G, Fuller R, Homer M, Robert T (2010). How to measure the quality of the OSCE: A review of metrics - AMEE guide no. 49. Med Teach.

[10] Khan KZ, Ramachandran S, Gaunt K, Pushkar P (2013). The Objective Structured Clinical Examination (OSCE): AMEE Guide No. 81 Part I: A historical and theoretical perspective. Med Teach.

[11] Chesser A, Cameron H, Evans P, Gleland J, Boursicot K, Mires G (2009). Sources of variation in performance on a shared OSCE station across four UK medical schools. Med Educ.

[12] Humphrey-Murto S, Touchi C, Wood TJ, Smee S (2009). Does the gender of the standardised patient influence candidate performance in an objective structured clinical examination?. Med Educ.

[13] Harasym PH, Woloschuk W, Cunning L (2008). Undesired variance due to examiner stringency/leniency effect in communication skill scores assessed in OSCEs. Adv Health Sci Educ Theory Pract.

[14] Turner JL, Dankosko ME (2008). Objective structured clinical exams: A critical review. Fam Med.

[15] Schultz JH, Nikendei C, Weyrich P, Möltner A, Fischer MR, Jünger J (2008). Qualitätssicherung von Prüfungen am Beispiel des OSCE-Prüfungsformats: Erfahrungen der Medizinischen Fakultät der Universität Heidelberg. Z Evid Fortbild Qual Gesundhwes.

[16] Barman A (2005). Critiques on the objective structured clinical examination. Ann Acad Med Singapore.

[17] Sloan DA, Donelly MB, Schwartz RW, Strodel WE (g). The Objective Structured Clinical Examination. The new gold standard for evaluating postgraduate clinical performance. Ann Sur.

[18] Mash B (2007). Assessing clinical skill - standard setting in the objective structured clinical exam (OSCE). South Afr Fam Pract.

[19] Jünger J (2018). Kompetenzorientiert prüfen im Staatsexamen Medizin. Bundesgesundheitsbl.

[20] Yeates P, O'Neill P, Mann K, Eva KW (2013). 'You're certainly relatively competent': Assessor bias dur to recent experiences. Med Educ.

[21] Iramaneerat C, Yudkowsky R (2007). Rater errors in a clinical skills assessmant of medical students. Eval Health Prof.

[22] Schleicher I, Leitner K, Juenger H, Moeltner A, Ruesseler M, Bender B, Sterz J, Schuettler KF, Koenig S, Kreuder JG (2017). Examiner effect on the objective structured cliniclal exam - a study at five medical schools. BMC Med Educ.

[23] Nikendei C, Kraus B, Lauber H, Schrauth M, Weyrich P, Zipfel S, Jünger J (2007). An innovative model for teaching complex clinical procedures: Integration of standardised patients into ward round training for final year students. Med Teach.

[24] Rethans JJ, Grosfeld FJ, Aper L, Reniers J, Westen JH, van Wijngaarden JJ, van Weel-Baumgarten EM (2012). Six formats in simulated and standardized patients use, based on experiences of 13 undergraduate medical curricula in Belgium and the Netherlands. Med Teach.

[25] Barrows HS (1993). An Overview of the uses of standardized patients for teaching and evaluating clinical skills. Acad Med.

[26] Schulz JH, Schönemann J, Lauber H, Nikendei C, Herzog W, Jünger J (2007). Einsatz von Simulationspatienten im Kommunikations- und Interaktionstraining für Medizinerinnen und Mediziner (Medi-KIT): Bedarfsanalyse - Training - Perspektiven. Gruppendyn Organisationsberat.

[27] Yeates P, O'Neill P, Mann K, Eva K (2013). Seeing the same thing differently - Mechanisms that contribute to assessor differences in directly-observed performance assessments. Adv Helath Sci Educ Theory Paract.

